# Why do children and adolescents (not) seek and access professional help for their mental health problems? A systematic review of quantitative and qualitative studies

**DOI:** 10.1007/s00787-019-01469-4

**Published:** 2020-01-21

**Authors:** Jerica Radez, Tessa Reardon, Cathy Creswell, Peter J. Lawrence, Georgina Evdoka-Burton, Polly Waite

**Affiliations:** 1 School of Psychology and Clinical Language Sciences, University of Reading, Reading, UK; 2 Departments of Experimental Psychology and Psychiatry, University of Oxford, Oxford, UK; 3 School of Psychology, University of Southampton, Southampton, UK; 4 Slough Community Mental Health Team, Berkshire Healthcare NHS Foundation Trust, Slough, UK

**Keywords:** Children, Adolescents, Mental health, Barriers, Facilitators, Professional help

## Abstract

Mental health disorders in children and adolescents are highly prevalent yet undertreated. A detailed understanding of the reasons for not seeking or accessing help as perceived by young people is crucial to address this gap. We conducted a systematic review (PROSPERO 42018088591) of quantitative and qualitative studies reporting barriers and facilitators to children and adolescents seeking and accessing professional help for mental health problems. We identified 53 eligible studies; 22 provided quantitative data, 30 provided qualitative data, and one provided both. Four main barrier/facilitator themes were identified. Almost all studies (96%) reported barriers related to young people’s individual factors, such as limited mental health knowledge and broader perceptions of help-seeking. The second most commonly (92%) reported theme related to social factors, for example, perceived social stigma and embarrassment. The third theme captured young people’s perceptions of the therapeutic relationship with professionals (68%) including perceived confidentiality and the ability to trust an unknown person. The fourth theme related to systemic and structural barriers and facilitators (58%), such as financial costs associated with mental health services, logistical barriers, and the availability of professional help. The findings highlight the complex array of internal and external factors that determine whether young people seek and access help for mental health difficulties. In addition to making effective support more available, targeted evidence-based interventions are required to reduce perceived public stigma and improve young people’s knowledge of mental health problems and available support, including what to expect from professionals and services.

**Electronic supplementary material:**

The online version of this article (10.1007/s00787-019-01469-4) contains supplementary material, which is available to authorized users.

## Introduction

Almost one in seven young people meet diagnostic criteria for a mental health disorder [1]. Untreated mental health disorders in children and adolescents are related to adverse health, academic and social outcomes, higher levels of drug abuse, self-harm and suicidal behaviour [2–4] and often persist into adulthood [5]. Indeed, half of the lifetime mental health problems start by the age of 15 and nearly three quarters by the age of 18 [6], creating a substantial global socioeconomic burden [7]. These short and longer term negative outcomes associated with youth mental health problems emphasise the importance of early detection and prompt access to professional treatment.

Effective, evidence-based treatments for mental health disorders in young people exist [8]. However, less than two-thirds of young people with mental health problems and their families access any professional help [9]. In general, young people are more likely to get professional help if they are older (i.e. adolescents more likely than children), Caucasian, experiencing more than one mental health problem and suffering from behavioural rather than emotional disorders [10, 11]. Besides from factors associated with treatment utilisation (e.g. gender and race), a detailed understanding of the reasons that young people (rather than parents or professionals) do not seek and access professional help is crucial to address the gap between the high prevalence of mental health disorders in young people and low treatment utilisation. A recent systematic review of parent-reported barriers to accessing professional help for their child’s mental health problems identified barriers related to systemic/structural obstacles (e.g. costs, waiting times), attitudes towards the service providers and psychological treatment (e.g. trust and confidence in professionals, the perceived effectiveness of treatment), knowledge and understanding of mental health problems and the help-seeking process (e.g. recognition of the problem, knowing where to get help) and family circumstances (e.g. other responsibilities and family’s support network) [12]. Amongst general practitioners (GPs), who often act as ‘gatekeepers’ between families and specialist services, commonly perceived barriers include difficulties identifying and managing mental health problems (e.g. confidence, time, lack of specific mental health knowledge) and making successful referrals for treatment (e.g. lack of providers and resources) [13].

As young people can take an active role in help-seeking, particularly as they get older, it is important to ascertain their own views on the barriers to seeking and accessing help for their mental health problems. A previous systematic review that focused on young people’s views found that young people most commonly fail to seek help because of stigma, embarrassment, difficulties with recognising problems and a desire to deal with difficulties themselves [14]. However, this review only considered help-seeking for anxiety, depression and general ‘mental distress’ and, therefore, does not capture barriers in the context of other mental health disorders, or more recent literature published since 2009. Furthermore, the review included samples of young adults (e.g. university students), making it hard to know the degree to which the reported barriers/facilitators are relevant for children and adolescents specifically.

It is now widely recognised that high demands on specialist services, limited available provision and long waiting lists present key barriers to accessing child and adolescent mental services [15]. This has prompted a range of recent initiatives designed to increase the availability and accessibility of specialist services (e.g. Children and Young People’s Improving Access to Psychological Treatment (CYP-IAPT) Programme in the UK, KidsMatter in Australia), support within schools [16, 17], and public resources (e.g. YoungMinds, ReachOut). However, it is critical that efforts to improve access to support and services consider young people’s views on help-seeking, and by doing so address the barriers that are pertinent to them.

This study provides an up-to-date systematic review of all studies where children and adolescents were asked about barriers and facilitators to help-seeking and accessing professional support in relation to a wide range of mental health difficulties, to inform ongoing and future interventions designed to improve treatment access. To fully address the complexity of the process of seeking and accessing professional help in young people, results from quantitative and qualitative studies were analysed and combined. By focusing on children and adolescents with a mean age of 18 years or younger (and excluding any studies which only included young adults over 21 years) findings will be especially relevant to the school context, and youth services for under 19s.

## Methods

A systematic review was conducted following PRISMA guidelines [18] and was registered in the international prospective register of systematic reviews (PROSPERO), number 42018088591, on 13/02/2018. A PRISMA checklist is provided in Online Resource 1.

### Literature search

The initial search strategy and preliminary inclusion/exclusion criteria followed a recent review of parent-perceived barriers and facilitators to help-seeking and accessing treatment for their children [12]. The search terms captured four major concepts: (1) barriers/facilitators, (2) help-seeking/accessing, (3) mental health, and (4) children/adolescents and parents (see Online Resource 2 for details of the search strategy). The original search was launched in October 2014 [12] and replicated using the same strategy in October 2016 and in February 2018. We used the NHS Evidence Healthcare database, combining Medline, PsycINFO and Embase, and the Web of Science Core Collection separately. Additionally, we used hand-search methods to check the reference list of articles included in the full text screening stage, and performed backward and forward reference searching of key papers to identify further studies of interest.

### Eligibility criteria

A study was included if child and/or adolescent (mean sample age up to 18) participants reported barriers and/or facilitators to seeking and accessing professional help for mental health problems. Studies reporting only parental/caregiver’s perceived barriers and facilitators, and studies including only young adults (e.g. university students) were excluded. Similarly, studies that only reported factors associated with treatment utilisation and studies reporting barriers/facilitators related to ongoing treatment engagement (not initial access to treatment) were excluded. The full list of inclusion/exclusion criteria is available in Online Resource 3.

### Study selection

We selected the studies for the current review through an initial search in October 2014 conducted within the Reardon et al. [12] review, and two updated searches using the same search terms (October 2014–October 2016; and October 2016–February 2018). In total, 3682 studies published since October 2014 were identified from database searches and hand searching. After duplicates were removed, two independent reviewers from the team (JR, CT, GEB, and PL) screened 2582 abstracts, and 385 full texts. In cases of disagreement, a third reviewer was consulted (TR) to reach a final decision. In total, 53 studies were included in the current review. Thirty studies provided qualitative data, 22 provided quantitative data and one study provided both. For two included studies, relevant results were reported in two separate papers, which were all included in a current review [19–22].

**Fig. 1:**
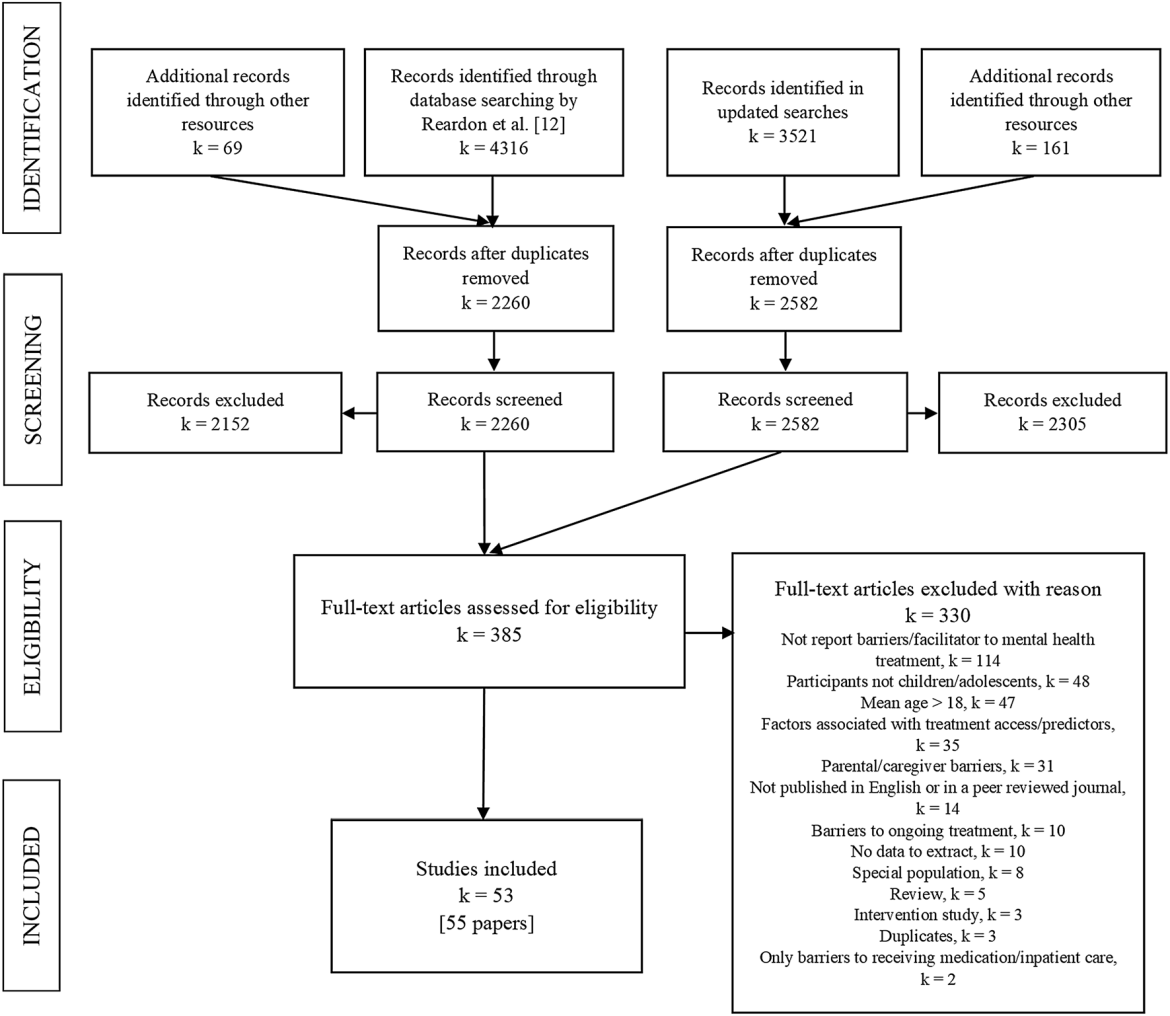
PRISMA flowchart of study selection process

The full process of study selection is presented in the PRISMA flowchart (Fig. 1).

### Data extraction

We used the data extraction form developed by Reardon et al. [12], with minor amendments to reflect the fact that study participants were children/adolescents rather than parents. The form included the following information: (1) methodology used (qualitative, quantitative or mixed methods), (2) country of study, (3) study setting (e.g. school, mental health clinic), (4) child/adolescent characteristics, including age range, gender, ethnicity, area of living (e.g. rural, urban), (5) type of mental health problem addressed/focus of the study and method of mental health assessment, (6) characteristics related to service use, and (7) key findings relating to perceived barriers and facilitators, supported by quantitative or qualitative evidence. Where applicable, details regarding barrier/facilitator measures were recorded for quantitative studies. For qualitative studies, we recorded details about the methods used (e.g. focus groups, interviews) and the areas of relevant questioning. Data extraction was undertaken by two independent reviewers (JR and GEB/PL/TR).

### Quality rating

In line with the approach used by Reardon et al. [12], we used two adapted versions of quality rating checklists developed by Kmet et al. [23]. One checklist was used to evaluate the quality of quantitative studies and another to evaluate the quality of qualitative studies. Quality checklists addressed the research question, study design, sampling strategy and data analysis. The quantitative checklist also addressed the robustness of the barrier/facilitator measure, and the qualitative checklist addressed the credibility of the study’s conclusions (see Online Resource 4). The quality of the study that provided qualitative and quantitative data [24] was assessed using both scales. Two independent reviewers (JR and GEB/PL/TR) assessed the quality of each included study. Based on the total score, each study was classified as ‘low’ (0–12 for quantitative and 0–11 for qualitative studies), ‘moderate’ (13–16 for quantitative and 12–15 for qualitative studies) or ‘high’ (17–20 for quantitative and 16–18 for qualitative studies) quality. Discrepancies between the reviewers were discussed with a third reviewer (TR/CC). Each study was included in the review, regardless of its quality.

### Data synthesis

We conducted a narrative synthesis following ESRC guidance [25], which outlines three main steps of analysis: (1) developing a preliminary synthesis, (2) exploring relationships between and within studies, and (3) assessing robustness of the synthesis. We chose this approach because of the high methodological variability across studies and the predominantly descriptive nature of the results. Consequently, statistical meta-analysis was not feasible.

A preliminary synthesis was done separately for quantitative and qualitative studies Each individual perceived barrier or facilitator reported in each quantitative study was assigned a code, and we reorganised the data according to these initial codes (e.g. ‘assured confidentiality’, ‘concerns around confidentiality’, ‘worrying that information about me will be shared with others’). We then used an iterative process to refine codes, to group codes into families of codes (e.g. ‘perceived confidentiality’), and finally to group families of codes into overarching barrier/facilitator themes (e.g. ‘relationship factors’). Extracted qualitative data were coded and organised following the same procedure. Next, we developed a single-coding framework capturing barriers and facilitators across quantitative and qualitative studies. Codes generated in the preliminary synthesis of qualitative and quantitative studies were combined and refined in this step, and organised into 22 subthemes and 4 themes. To address the heterogeneity of the quantitative studies and to facilitate comparison across studies, we ‘transformed’ the data [25]. In line with the ESRC guidance, we developed a ‘common rubric’ to summarise the quantitative data. After examining the percentages of participants who endorsed each specific barrier/facilitator across studies, we categorised each barrier/facilitator into one of three groups [‘low’ (endorsed by 0–10% of participants), ‘medium’ (endorsed by 10–30% of participants) and ‘high’ (endorsed by more than 30% of participants)]. These groups reflect the relative distribution of the percentage of respondents who endorsed each barrier/facilitator across studies. Where applicable, Likert-scale responses were converted into ‘percentage endorsed’ by summing positive responses (e.g. ‘agree’ and/or ‘strongly agree’) before categorisation. Three studies reported only means and standard deviations for each barrier/facilitator and no frequencies. In these cases, we applied data standardisation and categorised responses into the three corresponding categories using percentile and *z* scores. To minimise the impact of barriers/facilitators reported by only a small minority (< 10%) of participants, barriers/facilitators categorised as ‘low’ frequency were not included in subsequent analyses. As results from qualitative studies were descriptive (non-numerical), this kind of data transformation was not appropriate for qualitative studies.

We used graphical methods to present the percentage of included studies that reported each specific barrier/facilitator, and the corresponding percentage for qualitative and quantitative studies separately. Next, we explored the relationship between study characteristics (e.g. qualitative/quantitative methodology, country, use of a mental health assessment to identify participants) and sample characteristics (e.g. mental health status, gender, area of living), and barrier/facilitator themes and subthemes. Where we identified a pattern related to study/sample characteristics, details are reported below.

We performed a sensitivity analysis to establish the review’s robustness by examining the impact of ‘low’ quality studies on the findings. These studies were removed and results related to themes, subthemes and conclusions re-examined to determine whether they stayed the same or not.

All analyses were led by the primary author (JR), with regular discussions with other reviewers (TR/PW/CC) to agree with the interpretation of codes and themes.

## Results

### Study description

In total, 53 studies were included in the review, with 22 providing quantitative data, 30 providing qualitative data, and 1 study providing both [24]. Therefore, the total number of studies and corresponding percentages in the results refer to 54 included samples (23 quantitative and 31 qualitative). Study characteristics are provided in Tables 1 and 2.

**Table 1:** Characteristics of quantitative studies

References	Number of participants reporting barriers/facilitators	Age (range)	Country	Ethnicity	Females (%)	Area of living	Setting	Focus of the study	Mental health assessment	Source of professional help	Service use	Barrier/facilitator measure—details	Quality assessment
Boyd et al. [26]	201	11–18	Australia	No information	63%	Rural	School	Anxiety and Depression	CES-D SAS^a^	Any professional help	No information	Open-ended question about barriers to seeking professional help	17 (high)
Chandra and Minkovitz [32]	274	13–14	USA	46% white, 27.4% black, 9.5% Asian, 4.7% Hispanic and 9% multiracial	50%	Suburban	School	Emotional concerns	Not assessed	Mental health services	15.9% received psychological services or counselling (past year)	Ten barriers to help-seeking and ‘no barriers’ option	17 (high)
Cigularov et al. [33]	854	14–18	USA	78.3% Caucasian, 10.2% Hispanic, 2% Asian/Pacific, 1.7% Native American, 0.8% African American, 6.6% Other	47%	No information	School	Depression and suicidality	Not assessed	Any professional help	No information	26 barriers to help-seeking; 6-point Likert scale	18 (high)
D’Amico et al. [29]	2883	14–18	USA	69% Caucasian, 13% Hispanic, 5% Asian American/Pacific Islander, 2% American Indian/Alaskan Native, 1% African American, 10% ‘other’	50%	No information	School	Alcohol related problems and concerns	Adapted questionnaire to assess drinking habits (6.7% ‘heavy drinkers’ and 21% ‘problem drinkers’)	Alcohol related services	No information	14 facilitative factors; 5-point Likert scale	16 (moderate)
Freedenthal and Stiffman [27]	101 (out of 356 screened for suicidal thoughts)^b^	15–21	USA	American Indians	72%	51.5% lived on the reservation, 48.5% in urban areas	Participant home	Suicidality	Questions about suicidality (100% ever thought of/attempted suicide; 59.4% suicidal thoughts, 24% multiple suicide attempts, 10% one suicide attempt, 6% number of attempts not given), YSR	Any professional help	40.59% saw a MH professional; 12.87% consulted a school counsellor or teacher.	Open-ended question about barriers to seeking professional help with suicidal thoughts or behaviour.	17 (high)
Gould et al. [34]	519	13–19	USA	78% white, 3% African American, 13% Hispanic, 1% Asian, 4% other	50%	Urban and rural	School	Feeling very upset, stressed or angry	BHS (10% above threshold) CIS (28% above threshold)	Any professional help including hotlines	Hotline/substance programme/other health professional: 1.7–3.1 (last year), 2.1–3.3 (ever): School counsellor/ MH professional: 21.1–22-3(last year), 29.5–29.8 (ever)	16 barriers to help-seeking	17 (high)
Gould et al. [35]	24 (out of 317 identified ‘at risk’ and who did not seek help after referral at baseline)^b^	13–19	USA	58.3% white, 20.8% Hispanic, 12.5% Asian, 8.3% other	54%	No information	School	Suicidality	SIQ-JR (33% serious suicidal ideation), seven questions about suicide attempt history (25% past suicidal attempts), BDI-IA (58.3% depression), DUSI, CIS (37.5%)^c^	Mental health services	None	HUQ	17 (high)
Guo et al. [36]	865 Latin American	*M* = 12.6, SD = 1.96	USA	Latin American	51%	No information	School	Internalising and externalising problems	SDQ (20.3% elevated symptoms) and CIS	SBMHS	12.9% referred to SBMHS (past academic year)	Nine reasons for not seeking help	15 (moderate)
	936 Asian American			Asian American					SDQ (13.9% elevated symptoms) CIS		3.2% referred to SBMHS (past academic year).		
Guterman et al. [37]	858 Arab	14–17	Israel	46.7% Arab and 53.3% Jewish	57.9%	No information	School	Emotional distress—exposure to community violence	Adapted version of My ETV (87% witnessed ≥ 1 act of community violence (past year)	Any Professional Help	11.5% sought help from MH professional, 10% from youth group or religious leader, 9.2% from teacher and 8.7% from medical professional	16 reasons for not seeking help	18 (high)
	977 Jewish	14-17			54.5%				Adapted version of My ETV (92.5% witnessed ≥ 1 act of community violence (past year)		4.1% sought help from MH professional, 3.5% from youth group or religious leader, 2.7% from teacher and 2.1% from medical professional		
Haavik et al. [38]	1249	*M* = 17.6, SD = 1.15	Norway	No information	56%	Rural and urban	School	MH in general	Not assessed	MH Services	School-based MH services: 11–29.8%, Specialist MH services: 9.7–10.5%, Youth health station/GP: 16.2–32.9%	Nine barriers to help-seeking; 5-point Likert scale	18 (high)
Khairani et al. [39]	21 (out of 215 screened for depression symptoms)^b^	13–20	Malaysia	99.1% Malays, 0.9% Indians	57%	Rural	Primary care clinic	Depression	Structured self-report questionnaire with ten questions on depressive symptoms based on the DSM-IV (100% met criteria for depression)	Medical professionals	9.5% of those reporting significant depressive symptoms had sought medical help for these	No details about the barrier measure	15 (moderate)
Kuhl et al. [40]	280	14–17	USA	84% Caucasian, 9.8% Asian American, 4% Hispanic, 0.4% African American, 2.1% no ethnicity specified.	50%	No information	School	MH in general	50-item measure of physical and psychiatric symptoms developed by Dubow^a^	Any professional help	30% of participants were currently or previously in therapy	BASH	16 (moderate)
Lubman et al. [41]	2456	14–15	Australia	84.2% born in Australia; 1.9% in New Zealand, 1.4% in the United Kingdom, 1.1% in India and 1.0% in China	50%	Rural and urban	School	Depression and alcohol abuse; any professional help	Not assessed	Any professional help	30% sought help for MH problems from teachers or health professionals	BASH-B	20 (high)
Meredith et al. [24]	184 depressed^b^ 184 non-depressed^b^	13–17	USA	14.2% White, 32.7% Black, 49.3% Hispanic, 3.8% Other	78%	No information	Primary care clinic	Depression	DISC—depression module (100% of ‘depressed’ and 0% of ‘non depressed’ sample met the diagnostic criteria for depressive disorder in last 6 months)	Any professional help	55% reported receiving depression counselling (6 months after depression was identified) No information	Seven barriers; 5-point Likert scale	20 (high)
Muthupalani-appen et al. [42]	131 smokers 268 non-smokers	13–17	Malaysia	No information	No information	No information	School	Emotional and behavioural problems	YSR^a^	Primary care providers	5.3% sought help No information	16 reasons for not seeking help	15 (moderate)
Samargia et al. [43]	497 (those who reported having foregone mental health care from 878 screened)	16	USA	86.9% White, 0.9% African American, 3.2% Native American, 6.1% multiracial, 1.7% Asian, 1.1% Hispanic	65%	Rural and urban	School and community centres	Psychological or emotional problems	DHS and DHHS^a^	Mental health services	100%	11 reasons for not seeking help	17 (high)
Sharma et al. [44]	354	13–17	India	No information	48%	Urban	School	Depression	Not assessed	Any professional help	No information	No details about the barrier measure	9 (low)
Sheffield et al. [45]	254	15–17	Australia	89.7% Australian and 10.3% from other countries (mainly Asia, Europe, and the United Kingdom)	51%	No information	School	MH in general	The DASS-21^a^	Any professional help	9.1% sought help for a mental illness; 31.2% for a personal, emotional or behavioural problem (past 12 months)	Nine barriers to help-seeking	17 (high)
Sylwestrzak et al. [46]	10,123	13–18	USA	65.6% non-Hispanic whites, 15.1% non-His- panic blacks, and 14.4% Hispanics	49%	Rural and urban	Household and school	Emotional and behavioural problems and coping with stress	As a part of NCS-A Study they were asked about MH symptoms^a^	Any professional help	> 63% reported seeking treatment to manage and cope with emotions; 11.6% to help with controlling problem behaviours and 6.9% to help cope with stress	14 reasons for not seeking help	14 (moderate)
Wilson and Deane [47]	1037	13–21	Australia	95% Australian, remaining 5% European, Asian, North or South American, Middle Eastern, and ‘other’	59%	Urban	School and youth community groups	Psychological problems	Not assessed	MH Services	No information	BASH-B	18 (high)
Wilson et al. [28]	1184	11–17	Australia	No information	50%	Urban	School	Depression	CES-D (10.5% with moderate–severe depression symptoms)	MH Services	No information	Open-ended question about perceived barriers to seeking professional help	16 (moderate)
Wilson et al. [30]	173 (trial)	14–16	Australia	88% Australian, 6% European, 2% Asian	58%	No information	School	Psychological problems	Not assessed	Help-seeking with GPs	6.9–8.1% had ≥ 1 consultation with GP about psychological health	BETS	18 (high)
	118 (comparison group)			86% Australian, 9% European, 2% Aboriginal	60%						1.7–4.2% had ≥ 1 consultation with GP about psychological health		
Wu et al. [48]	119	7–13	USA	82% White, 12% African American, 3% Asian, 3% other	50%	No information	Community mental health centres	Paediatric anxiety	MASC, CAIS-C/P, CDI, PARS, ADIS-IV-C/P (66% GAD, 43% Social Phobia, 41% ADHD, 39% Separation Anxiety, 29% Specific Phobia)	MH Services	100%	TAQ-CA	20 (high)

*CAIS-C/P* Child Anxiety Impact Scale-Child/Parent Report, *CDI* Children’s Depression Inventory, *CES-D* Centers for Epidemiologic Studies Depression Scale, *CIS* Columbia Impairment Scale, *DASS-21* Depression Anxiety Stress Scale, *DHS* Minnesota Students Survey, *DHSS* Youth Risk Behaviour Survey, *DISC* Diagnostic Interview Schedule for Children, *DUSI* Drug Use Screening Inventory, *HUQ* Help-Seeking Utilization Questionnaire, *MASC* Multidimensional Anxiety Scale for Children, *MH* mental health, *My ETV* My Exposure to Violence Scale, *PARS* Pediatric Anxiety Rating Scale, *SAS* Self-rating Anxiety Scale, *SBMHS* school-based mental health services, *SDQ* Strengths and Difficulties Questionnaire, *SIQ-JR* Suicidal Ideation Questionnaire, *TAQ-CA* Treatment Ambivalence Questionnaire-Child (Anxiety) Version, *YSR* Youth Self-Report

^a^Results of MH assessment not reported. *ADIS-IV-C/P* Anxiety Disorders Interview Schedule for DSM-IV-Child and Parent Versions, *BASH* Barriers to Adolescents Seeking Help, *BASH-B* the brief version of the Barriers to Adolescents Seeking Help scale

^b^Mental health assessment used to identify participants. *BETS* Barriers to Engagement in Treatment Screen, *BDI-IA* Beck Depression Inventory, *BHS* Beck Hopelessness Scale

^c^The same methods were used in baseline screening

**Table 2:** Characteristics of qualitative studies

References	Number of participants reporting barriers/facilitators	Age (range)	Country	Ethnicity	Females (%)	Area of living	Setting	Focus of the study	Mental health assessment	Source of professional help	Service use	Barrier/facilitator measure—details	Quality Assessment
Balle Tharaldsen et al. [49]	8	17–18	Norway	88.5% Norwegian, 12.5% immigrant background.	75%	No information	School	MH in general	No previous MH problems^b^	Any professional help	0% current, 25% previous contact with professional help.	Interviews	17 (high)
Becker et al. [50]	13	12–17	USA	Majority Caucasian	38%	No information	Community—outreach and support programmes for military families	MH in general	Not assessed	MH services	No information	Interviews and focus groups	16 (high)
Breland-Noble et al. [51]	29	11–17	USA	African American	No information	Rural, urban and suburban	No information.	Depression	Not assessed	Any professional help	No information	Interviews and focus groups	12 (moderate)
Bullock et al. [52]	15	14–18	Canada	100% Canadian with heterogeneous ethnicities (i.e. mixed European, Aboriginal). One youth was a 2nd generation migrant.	87%	No information	The Depressive Disorders (outpatient) Program of a psychiatric hospital	Suicidality	K-SADS-PL, SCID-II (53% depressive disorder, 33% cluster B personality disorder) questions about suicidality (60% lifetime suicide attempt, 40% multiple attempts)	Any professional help	100%	Interviews	12 (moderate)
Bussing et al. [31]	148	14–19	USA	No information	59%	Rural and urban	School	ADHD	Screening questions with parents and CASA (74% ADHD high risk).	Psychiatric/medical and psychological help	57% had a previous ADHD treatment	Open-ended survey questions	14 (moderate)
Chandra and Minkovitz [53]	57	13–14	USA	30% African American	74%	Suburban	School	MH in general	Not assessed	MH services	38.6% within and 10.5% outside the school	Interviews	16 (high)
Clark et al. [54]	29	12–18	Australia	No information	0%	Rural and urban	School and MH Clinic	Clinical anxiety	28% history of anxiety diagnosis^b^	Any professional help	28% had previously sought professional help	Interviews and focus groups	17 (high)
De Anstiss and Ziaian [55]	85	13–17	Australia	100% refugees of mixed ethnic backgrounds	56%	No information	Community—refugee centres, programmes, schools	MH in general	Not assessed	MH services	Not clear	Focus groups	15 (moderate)
Del Mauro and Jackson Williams [56]	31	7–18	USA	87.1% Caucasian, 3.2% African American, 3.2% Asian, 3.2% Hispanic, 3.2% not reported	71%	Rural and urban	Community	MH in general	Not assessed	Psychological help	19.4% ≥ 1 therapy session	Focus groups	15 (moderate)
Doyle et al. [57]	35	16–17	Ireland	No information	66%	Urban	School	MH in general	Not assessed	Help-seeking in schools	No information	Focus groups	12 (moderate)
Fleming, Dixon and Merry [58]	39	13–16	New Zealand	49% Maori, 38% Pacific Islands, 10% New Zealand European	26%	No information	School—alternative schooling programmes for students excluded or alienated from mainstream education	Depression	Not assessed	Health providers and computer-based help	No information	Focus groups	16 (high)
Fornos et al. [59]	65	13–18	USA	89% Mexican–American	No information	Urban	School	Depression	Not assessed	Any professional help	No information	Focus groups	10 (low)
Fortune et al. [19, 20]	2954 (out of 6020 screened)^c^	15–16	UK	82% White, 12% Asian, 3% Black and 3% Other	54%	No information	School	Self-harm and suicidality	Questions about self-harm (10% lifetime self-harm) and scales to measure depression, impulsivity, anxiety and self-esteem^a^	Any professional help	No information	Open-ended survey questions	13 (moderate)
	332 who did not seek help before self-harm (out of 593 with a lifetime history of self-harm)^c^ 412 who did not seek help after self-harm (out of 593 with a lifetime history of self-harm)^c^	15–16	UK	88% White	Around 75%	No information	School	Self-harm	Questions about self-harm (100% lifetime history of self-harm) and scales to measure depression, impulsivity, anxiety and self-esteem^a^	Any professional help	14% got help before the episode of self-harm 1.4% got help after the episode of self-harm	Open-ended survey questions	13 (moderate)
Francis et al. [60]	52	14–16	Australia	No information	71%	Rural	School	MH in general	Not assessed	Any professional help	No information	Focus groups	16 (high)
Gonçalves et al. [61]	16	12–17	Portugal	25% Portuguese (African descendent)25% Cape Verde, 18.8% Brasil, 18.8% Angola, 12.5% Other	31%	No information	School	MH in general	Not assessed	Any professional help	31.3% had previous psychologist visit	Focus groups	14 (moderate)
Gronholm et al. [62]	29^c^	12.2–18.6	UK	65.5% White, 31% Black, 3.4% Asian	65.50%	Urban	School	Inter-/externalising disorder and risk of developing psychotic disorder	SDQ (100% borderline or clinical level of inter-/externalising disorder PLE (100% ≥ 1 ‘yes’ response to question regarding psychotic-like experiences)	Any professional help	No information	Interviews	18 (high)
Hassett and Isbister [63]	8^c^	16–18	UK	No information	0%	No information	Clinic—CAMHS	Self-harm and suicidality	≥ 2 episodes of self-harm (past 12 months)^b^	MH services	100%	Interviews	17 (high)
Huggins et al. [64]	6	18	USA	No information	No information	Rural and urban	School	MH in general	Not assessed	School-based mental health services	No information	Interviews	17 (high)
Kendal et al. [65]	23	11–16	UK	No information	65%	Urban	School	Emotional difficulties	Not assessed	School-based pastoral support	39% had used the MH service in school	Interviews	15 (moderate)
Klineberg et al. [66]	30	15–16	UK	40% Asian, 23% Black, 20% Mixed ethnicity, 13% White British and White Other	80%	Urban	School	Self-harm	Adapted version of A-Cope (33% never self-harmed, 30% self-harmed once, 37% more than once)	Any professional help	No information	Interviews	16 (high)
Leavey, Rothi and Paul [67]	48	14–15	UK	No information	50%	Urban	School	Emotional and mental health problems	Not assessed	Primary care providers	No information	Focus groups	11 (low)
Lindsey, et al. [68]	16	11–14	USA	100% African American	50%	Urban	School	MH in general	Not assessed	MH services	44% received school-based counselling	Focus groups	17 (high)
Lindsey et al. [21, 22]	18^c^	14–18	USA	100% African American	0%	Urban	Community based mental health centres and after-school programs for youth	Depression	CES-D (100% elevated levels of depression symptoms)	MH services	55% of them were in treatment	Interviews	16 (high)
Mcandrew and Warne [69]	7^c^	13–17	UK	100% White British	100%	No information	n/a	Self-harm and suicidality	100% experienced self-harm and/or suicidal behaviour^b^	Any professional help	No information	Interviews	13 (moderate)
Meredith et al. [24]	16^c^	13–17	USA	No information	No information	No information	Primary care clinic	Depression	Diagnostic Interview Schedule for Children—depression module (100% met criteria for depression)	Any professional help	Not clear	Interviews	10 (low)
Mueller and Abrutyn [70]	10	No information	USA	Upper middle class community	No information	Suburban	Community	Suicidality	Not assessed	Any professional help	No information	Interviews and focus groups	11 (low)
Pailler et al. [71]	60	12–18	USA	65% African American, 27% White, 8% multiracial	52%	Urban	Tertiary care children’s hospital	Depression	Not assessed	MH services	No information	Interviews	18 (high)
Prior [72]	8	13–17	UK (Scotland)	No information	75%	No information	School	MH in general	Not assessed	School counselling	100% received school counselling	Interviews	15 (moderate)
Timlin-Scalera et al. [73]	26	14–18	USA	100% White	15%	Suburban	School	MH in general	Not assessed	Any professional help	Not clear	Interviews	17 (high)
Wilson and Deane [74]	23	14–17	Australia	91% Australians of European descent, 4% Aboriginal, 4% Pakistani	52%	Urban	School	MH in general	Not assessed	Any professional help	No information	Focus groups	16 (high)
Wisdom et al. [75]	22	14–19	USA	90% White (non-Hispanic), other people with African, Hispanic, and Asian heritage	59%	No information	School and MH Clinic	Depression	Assessed by primary care providers^a^	Primary care providers	Not clear	Interviews and focus groups	16 (high)

*CASA* Child and Adolescent Services Assessment, *CES-D* Centers for Epidemiologic Studies Depression Scale, *K-SADS-PL* Schedule for Affective Disorders and Schizophrenia for School-Age Children-Present and Lifetime Version, *MH* mental health, *SCID-II* Structured Clinical Interview for DSM-IV Axis II

^a^Study does not provide results of MH assessment

^b^Study does not provide information on how MH was assessed

^c^Mental health assessment used to identify participants

Studies varied widely on sample size (from 6 to 10,123), participants’ age (from 7 to 21 years), country (with 48% of studies conducted in North America, 24% in Europe, 20% in Australia and 8% in Asia), demographic profiles (with 20% of studies focused on specific ethnic/gender groups and others with more varied samples), recruitment setting (with 72% of studies conducted in schools, 17% in (mental) health settings, and the others in varying community settings) and the type of mental health problem that was a focus of the study (with 30% of studies focused on mental health in general and the remaining studies focused on specific mental health problems, such as depression, anxiety, suicidal ideation and ADHD). In half of the studies participants’ mental health was assessed (all of these studies assessed young people’s mental health using questionnaire measures, with the exception of four studies that used a standardised diagnostic assessment). Similarly, studies addressed various types of professional support, with some (9%) focused on school-based (mental health) services and the majority of remaining studies focused on any professional help (50%) or on support available in a specific (mental) health setting (40%). In 41% of studies, participants’ service use was not reported or assessed, and in others, some (2–57%) or all participants had received professional help for their mental health problems.

In quantitative studies, young people were most commonly asked to endorse the presence or absence of barriers from a list, or rate barriers using a 4–7 point Likert response scale. Three quantitative studies asked open questions about help-seeking [26–28]. Less than a third (30%) of quantitative studies reported facilitators to help-seeking, with two of those studies reporting facilitators only [29, 30].

The majority of qualitative studies used one-to-one interviews (45%), focus groups (32%), or both (16%) to collect data, with the exception of two studies where they applied a qualitative approach to analyse responses to open-ended survey questions [19, 20, 31]. Unlike quantitative studies, more than a half (58%) of qualitative studies reported facilitators to help-seeking, as well as barriers.

### Quality ratings

Overall, the quality of the studies varied, ranging from ‘low’ to ‘high’, with 65% of quantitative and 52% of qualitative studies rated as ‘high’ quality, and 4% of quantitative and 13% of qualitative studies rated as ‘low’ quality. The weak aspects of qualitative studies tended to relate to methodological issues, such as clarity and appropriateness of sampling strategy (e.g. insufficient detail on how study participants were selected), data collection and analysis methods (e.g. only a very brief description of data analysis), whereas quantitative studies most commonly failed to describe the barrier/facilitator measure’s robustness (e.g. no details given about the measure’s psychometric characteristics).

### Barrier/facilitator themes

Four barrier/facilitator themes were identified from both the qualitative and quantitative studies. The themes relate to (1) young people’s individual factors, (2) social factors, (3) factors related to the relationship between the young person and the professional and (4) systemic and structural factors. Barrier and facilitator themes and subthemes are summarised below. Barrier and facilitator themes and subthemes identified in each study are available in Online Resource 5.

1. Young people’s individual factors

**Fig. 2:**
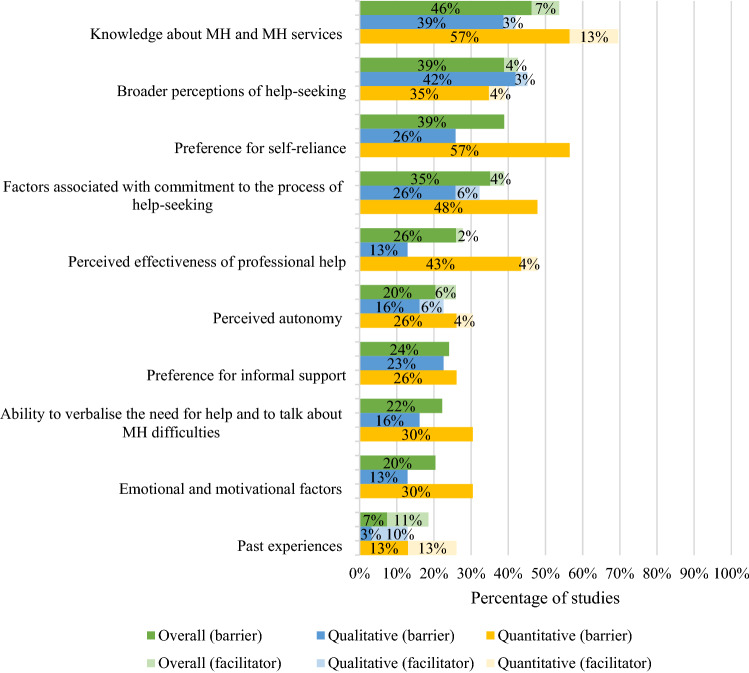
Percentage of all (percentage of 54 included samples that reported barrier/facilitator), qualitative (percentage of 31 included qualitative samples that reported barrier/facilitator) and quantitative (percentage of 23 included quantitative samples where a ‘large’ (> 30) or ‘medium’ (10–30) percentage of participants endorsed the barrier/facilitator) studies reporting barriers and facilitators relating to young people’s individual factors

The majority (96%) of studies reported barriers and facilitators related to individual factors. Subthemes and their distribution across all studies, and across qualitative and quantitative studies separately are outlined in Fig. 2.

Barriers and facilitators related to knowledge about mental health and mental health services were reported in more than half (53%) of the studies, and with high endorsement rates (> 30% of participants). Young people reported not knowing where to find help and/or whom to talk to [20, 34, 37, 38, 42–46, 64, 65, 69, 73, 74] and failing to perceive a problem as either serious enough to require help [20, 63] or mental health related [32]. Young people’s broader perceptions of help-seeking were reported as barriers in 39% of studies, and as facilitators in 4%. This subtheme captured young people’s general attitudes towards mental health and help-seeking [31, 49, 53, 55, 59], help-seeking expectations [20, 27, 31, 33, 37, 38, 46, 48, 54, 59, 68, 75] and perceptions about how help-seeking reflects on their character, such as perceiving help-seeking as a sign of weakness [21, 49, 54, 60, 63, 73, 75]. The latter was reported in all studies that included male-only samples. Young people commonly (in 39% of the studies) endorsed the barrier of refusing to seek help because of a desire to cope with their problems on their own [20, 21, 24, 26–28, 33, 34, 37, 40–42, 45–47, 50, 54, 56, 61, 68, 73]. This subtheme was reported in nearly all studies that included young people with elevated levels of depression symptoms or experiences of self-harm, and mostly in quantitative studies with high rates of endorsement. In 35% of the studies, young people reported barriers related to uncertainty about whether problems were serious enough to require help [34, 35, 37, 40, 42, 62, 66, 73, 74] and expectations that the problems would improve on their own [33–35, 40, 42, 43, 46]. Young people also endorsed barriers which related to a reluctance to attend appointments and adhere to recommended treatments [24, 71]. Factors associated with commitment to the process of help-seeking were usually endorsed with a high frequency within quantitative studies. Around a quarter of studies reported the perceived effectiveness of professional help to be the reason for (not) seeking professional help, with most studies reporting that young people were doubtful about the effectiveness of professional help [31–35, 37, 40, 42, 44–46, 48, 50, 67, 72]. This reason was endorsed by young people with or without previous experience of professional help. Notably, perceived effectiveness was more commonly reported in quantitative studies than qualitative studies. The extent to which young people perceive help-seeking as their own decision was reported in a quarter of the studies. Young people reported that they were more likely to seek help if they perceived it to be their own choice [65, 72] and less likely to seek help if they perceived it as their parents’/teachers’ choice [48, 61, 67]. A preference for informal support was reported as a barrier to seeking professional help in 24% of studies; young people reported that they would prefer to discuss their mental health difficulties with family members and friends than professionals [22, 26, 34, 40, 42]. The subtheme of young people’s ability to verbalise the need for help and to talk about mental health difficulties was the next most common barrier to help-seeking, and endorsed by young people in 22% of studies overall, and more commonly reported in quantitative than qualitative studies. One-fifth of the studies reported emotional and motivational factors related to the nature of their problem, such as anxiety [39–41, 43, 47, 69] and depression symptoms [20, 27, 33, 40], and a lack of motivation [54, 58] as barriers to seeking professional help. Unsurprisingly, anxiety and depression symptoms were most frequently reported as posing barriers in the studies that included participants with elevated levels of psychological distress. This subtheme only captured barriers and was more frequently reported in the quantitative studies than qualitative studies. Young people also reported past experiences to be both facilitators [26, 40, 47, 53, 73, 74] and barriers [35, 40, 46, 53] to seeking professional help for their mental health problems. Past positive experience was the most commonly reported facilitator, reported in 15% of studies.

2. Social factors

**Fig. 3:**
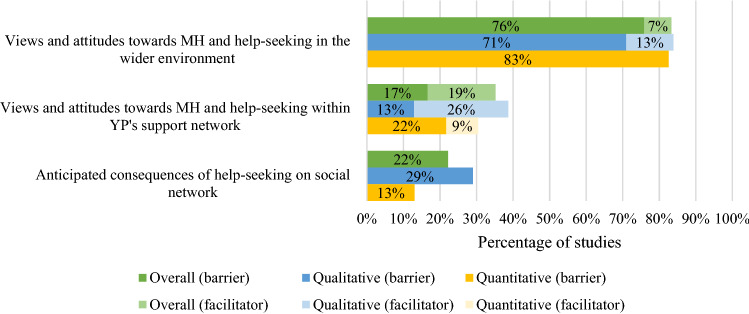
Percentage of overall (percentage of 54 included samples that reported barrier/facilitator), qualitative (percentage of 31 included qualitative samples that reported barrier/facilitator) and quantitative (percentage of 23 included quantitative samples where a ‘large’ (> 30) or ‘medium’ (10–30) percentage of participants endorsed the barrier/facilitator) studies reporting barriers and facilitators relating to social factors

The second theme describes barriers and facilitators related to social factors and this theme was reported in 92% of studies. Subthemes in this category are outlined in Fig. 3.

The vast majority of studies reported barriers (76% of studies) related to perceived stigma [19–21, 26, 27, 31, 32, 49, 50, 54–62, 64, 68, 69, 72, 73] and young people’s experienced and/or anticipated embarrassment as a consequence of negative public attitudes [20, 22, 27, 28, 32, 33, 36, 37, 40–42, 44, 47–49, 58, 61, 64, 69], and these barriers were usually reported by a high percentage of young people within studies. Reduced public stigma and public normalisation of help-seeking were reported as related facilitators in four (13%) qualitative studies [57, 63, 72, 74]. Views and attitudes towards mental health and help-seeking within young people’s support networks, such as family, friends, teachers and GPs, were reported as barriers in 17% of studies, and as facilitators in 19% of studies. In most of these studies, these barriers/facilitators were reported by a high percentage of participants. Notably, positive views and encouragement from young people’s support networks were commonly reported facilitators (26% of qualitative and 9% of quantitative studies) [21, 32, 52, 59, 61, 63, 72, 73]. This subtheme was more frequently reported in studies including ethnically diverse samples, ethnic minorities or only male participants than studies with predominantly Caucasian, and mixed-gender samples. Anticipated consequences of help-seeking on young people’s social network included the fear of being taken away from their parents [59], fear of losing status in a peer group [49] and making their family angry or upset [48] and were reported as barriers in 29% of qualitative and 13% of quantitative studies.

3. Relationship factors

**Fig. 4:**
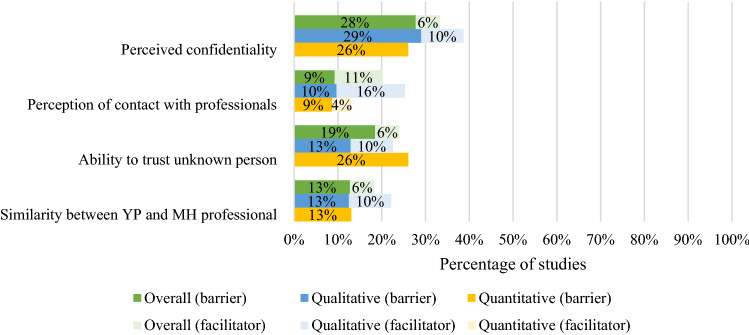
Percentage of overall (percentage of 54 included samples that reported barrier/facilitator), qualitative (percentage of 31 included qualitative samples that reported barrier/facilitator) and quantitative (percentage of 23 included quantitative samples where a ‘large’ (> 30) or ‘medium’ (10–30) percentage of participants endorsed the barrier/facilitator) studies reporting barriers and facilitators relating to relationship factors

A large proportion of studies (68%) reported barriers and facilitators related to the relationship between the young person and a mental health professional. The distribution of subthemes across studies overall, and among qualitative and quantitative studies, is outlined in Fig. 4.

Issues related to perceived confidentiality were reported as barriers in 28% and facilitators in 6% of the studies [19, 29, 33, 36, 37, 39, 45, 47, 50, 56, 57, 59, 62, 64–66, 69, 73, 74]. Young people also reported concerns regarding disclosing personal information to a person they do not know well [22, 26, 28, 32, 33, 35, 42, 57, 58, 65, 68, 72, 74]. Barriers and facilitators related to young people’s perceptions of contact with professionals were reported in one-fifth of the studies (20%). Young people reported that they are more likely to seek help if they feel respected [63, 66], listened to [29, 30, 69] and not judged [69], and less likely if they feel they are being judged or not taken seriously [20, 37, 38, 56, 69]. Lastly, young people endorsed barriers and facilitators related to similarities/differences between them and professionals in 13% and 6% of studies, respectively. This subtheme was most frequently reported in qualitative studies that included ethnically diverse samples, ethnic minorities and only male participants, and included references to the gender [63], ethnicity/race [21] and age [40, 47] of professionals.

4. Systemic and structural factors

**Fig. 5:**
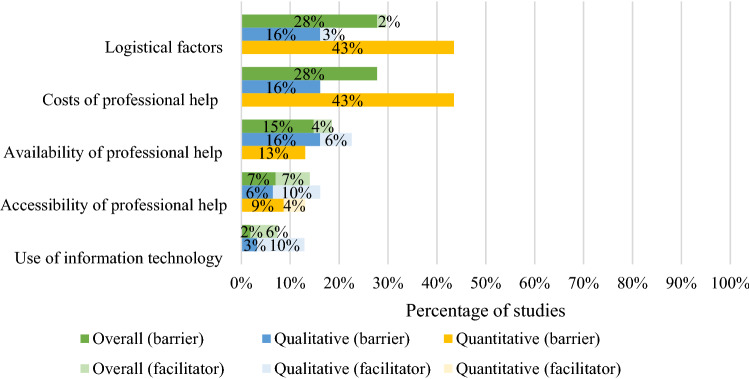
Percentage of overall (percentage of 54 included samples that reported barrier/facilitator), qualitative (percentage of 31 included qualitative samples that reported barrier/facilitator) and quantitative (percentage of 23 included quantitative samples where a ‘large’ (> 30) or ‘medium’ (10–30) percentage of participants endorsed the barrier/facilitator) studies reporting barriers and facilitators relating to systemic and structural factors

Barriers and facilitators related to systemic and structural factors were reported by 58% of studies overall. We identified six subthemes which are outlined in the Fig. 5.

Logistical factors, such as lack of time [24, 35, 40, 42], interference with other activities [24, 48], transportation difficulties [36, 42, 45] and costs associated with mental health services [24, 31, 35, 36, 38, 40–43, 45, 46, 50, 59, 61, 71] were reported in a large proportion of studies, and predominantly in quantitative studies. Two-thirds of studies reporting costs as a barrier to professional support were American and studies reporting transportation difficulties were more commonly conducted in rural areas than in cities. Young people also frequently reported barriers (15% of the studies) and facilitators (4% of the studies) related to the availability of professional help. Limited availability of professional services and excessive waiting times were the most commonly reported barriers within this subtheme [24, 26, 38, 54, 60, 68, 73]. Studies also reported barriers related to difficulties accessing or reaching support, for example, difficulties making an appointment or the attitude of staff towards them [19, 24, 33, 67]. The last subtheme captured young people’s perceptions of the role of information technology in help-seeking. In 10% of qualitative studies, young people identified opportunities to communicate distress and attend treatment via digital tools as facilitators to seeking/accessing treatment [54, 58, 63, 67]. All of these studies were conducted in the UK, Australia or New Zealand.

### Robustness of data synthesis

Sensitivity analysis was performed by excluding four ‘low quality’ studies (three qualitative and one quantitative) and re-examining the distribution of themes and subthemes among the remaining studies. There was minimal change in relation to the distribution of barrier/facilitator subthemes across qualitative and quantitative studies, and the overall results remained similar and conclusions unchanged.

## Discussion

This review identified 53 studies addressing barriers and facilitators to seeking and accessing professional help for mental health problems as perceived by children and adolescents. We identified four themes across the studies. Barriers and facilitators related to young people’s individual factors and to social factors were identified in the vast majority of the studies. Young people also commonly reported barriers and facilitators related to the relationship between them and professionals and to systemic and structural factors.

Among barrier/facilitator subthemes, young people most frequently endorsed barriers and facilitators related to societal views and attitudes towards mental health and help-seeking, such as perceived public stigma and embarrassment associated with mental health problems. Young people also often perceived a lack of knowledge about mental health and the available help as a barrier to help-seeking. Young people with a prior experience of mental health difficulties reported that, during their difficulties, they had not recognised the need for professional help and had not perceived their problems as not serious enough to require help. Young people’s negative expectations and attitudes towards professionals, and perceiving help-seeking as a sign of one’s weakness, were commonly reported across studies as well. The latter subtheme was almost always reported in studies which included only male participants, highlighting potential gender differences in perceived barriers [54]. Adolescents also often endorsed a preference to rely on themselves when facing mental health difficulties rather than seeking professional help, which was again especially prominent in studies where participants had previous experience of mental health difficulties. Notably, this subtheme was far more commonly reported in quantitative than qualitative studies. Compared to qualitative studies, quantitative studies also more commonly reported barriers and facilitators related to a commitment to the process of help-seeking, such as not perceiving a problem as serious enough and waiting for the problem to improve on its own. Lastly, the extent to which young people believed information shared between them and professionals would be treated as confidential seemed to play a significant role in whether young people decide to seek help or not.

This review’s findings are broadly consistent with the previous review by Gulliver and colleagues that focused on young people’s help-seeking for anxiety, depression and distress [14]. Our review makes a significant further contribution to the existing literature by including young people’s perceived barriers for a wider range of mental health difficulties. In line with our findings, Gulliver et al. [14] identified that the most common barriers and facilitators related to public, perceived and self/stigmatising attitudes, mental health knowledge, young people’s preference for self-reliance and perceived confidentiality. However, Gulliver et al. [14] reported that structural factors (e.g. logistical factors and costs related to professional help), anxiety symptoms, and characteristics of mental health service providers were more common than we found in this review. Furthermore, while Gulliver et al. [14] found that past positive experiences of help-seeking was the most frequently reported facilitator across studies, we found that (1) positive attitudes and encouragement from young people’s support network and (2) positive perceptions of the contact between them and professionals were the most commonly reported facilitators. These observed differences are likely to reflect the larger number of studies included in the current review than the previous review, with nearly two-thirds of included studies published since the review by Gulliver et al. [14]. Furthermore, the current review excluded studies with only young adult participants (e.g. university students), who may well perceive different barriers and facilitators to seeking help than younger adolescents.

### Implications

Our findings highlight many potential ways to improve access to treatment for young people experiencing mental health difficulties. First, the review highlights the ongoing need to minimise perceived mental health stigma among young people. There are a growing number of large-scale public health initiatives (e.g. *Time to Change* in the UK and *Opening Minds* in Canada) and school-based interventions [76] that are designed to reduce stigma and improve young people’s mental health and help-seeking literacy. Once the effectiveness of such programmes has been demonstrated, widespread dissemination is critical, making constructive conversations about mental health a part of the daily school routine. Our findings indicate that these interventions should focus on improving young people’s knowledge and understanding of mental health problems, [54], equipping young people with self-help skills and strategies [34], normalising mental health problems and the process of help-seeking [63, 74], ‘demystifying’ professional help [72], explaining which problems require help and which may not [20], and informing young people about where to find help and what to expect from it [30, 40], including explaining the therapeutic ‘ground rules’ (e.g. confidentiality). If we want to close the gap between high prevalence of mental health disorders and low treatment utilisation, sufficient service provision and professional support must be widely available for young people. Providing services within the school environment could address the systemic and structural barriers by minimising the effort required to access youth mental health services. Further, this could help reduce the barriers related to logistical factors, such as lack of time and transportation difficulties. Indeed, hundreds of schools in the UK already work collaboratively with local child and adolescent mental health services to offer specialist support and treatments to young people, teachers and parents at school [77]. With careful implementation, this may also be less stigmatising than a clinic environment [16], potentially helping greater numbers of young people to seek and access evidence-based treatments [78]. In addition, young people should be as equipped as possible to help themselves. Digital tools might be a means to increase access to support for mental health problems, and young people in studies in our review identified benefits of, for example, text messages [63, 67] to self-refer and to communicate with professionals directly Similarly, young people suggested using computerised psychological treatments [58], which might be especially appropriate for those who find it hard to talk about their feelings in person, and may help improve young people’s perceived independence. Equally, ensuring services are free at the point of use would minimise financial barriers to help-seeking/accessing. As young people’s support networks, especially families, seem to play the most important facilitative role in their process of help-seeking/accessing, professionals should be mindful about seeking appropriate family involvement, whilst balancing this against young people’s desire to make their own decisions about receiving help. It is clear that wherever interventions are provided, they must promise young people privacy [65] and promote their agency, control and self-determination [72].

### Strengths and limitations

This review provides a comprehensive overview of the most common reasons given by young people about why they may or may not seek and access professional help when experiencing mental health difficulties. The inclusion of qualitative studies provided additional contextual information and more detailed insight into young people’s experiences than was commonly captured in quantitative studies. By including all recent studies focusing on a wide range of mental health difficulties, it provides an update to and extension of the previous review published nearly a decade ago. Although the eligibility criteria for this review were narrower (i.e. excluding the studies with only young adults), there were twice as many studies included in this review as in the previous one, highlighting the rapid development of this field and the need for an updated review. Finally, the review was conducted using rigorous and systematic methodology. Nevertheless, the review has some limitations. Due to the high variability of included studies it was not possible to carry out detailed group comparisons in relation to the type of mental health problem, source of professional help, study setting and participants’ treatment utilisation. Furthermore, only four studies used a standardised diagnostic assessment to assess participants’ mental health, and many studies did not report/assess participants’ mental health at all, making it hard to perform reliable comparisons of findings among adolescents with different mental health problems. Another limitation relates to the fact that the review only includes studies published in English in peer-reviewed journals and, therefore, findings from studies published in other languages and in alternative publications were not captured here. Finally, it is important to acknowledge that the systematic search used to identify studies for inclusion in this review was conducted in February 2018 and, therefore, any relevant studies published since this date were not included in the review. Similar to previous research [12], our review identified that existing quantitative barrier/facilitator questionnaire measures are (1) more focused on barriers than facilitators and (2) tend to overlook some barriers/facilitators, especially those related to the role of young people’s support network and the characteristics of the relationship between young people and professionals. Results from the quantitative studies might, therefore, at least partly reflect the fact that young people were not asked about certain barriers and facilitators. These limitations of quantitative studies highlight the importance of including qualitative studies as well.

## Conclusions and further research

The main reasons for (not) seeking and accessing professional help given by young people are those related to mental health stigma and embarrassment, a lack of mental health knowledge and negative perceptions of help-seeking. Young people also reported a preference for relying on themselves when facing difficulties, and issues with committing fully to the process of help-seeking/accessing. Widespread dissemination of evidence-based interventions delivered in schools targeting perceived public stigma and young people’s mental health knowledge is needed. Furthermore, the collaboration between schools and mental health services is essential to enable young people and their families to access evidence-based support within settings that minimise the logistical barriers. Mental health professionals should also offer young people different ways to access help on their own, including using digital tools, which have a potential to facilitate help-seeking behaviour and promote young people’s agency.

Our review identified a few possibilities for further research. The lack of established self-report quantitative measures of barriers and facilitators of seeking and accessing mental health support for young people highlights the need to develop and evaluate a new questionnaire. Findings from the qualitative studies should be considered when revising the content of the existing questionnaire items to ensure all relevant barriers/facilitators are captured, and their prevalence can be established. To inform mental health services for specific disorders in children and young people, studies examining barriers and facilitators to seeking and accessing professional help for children and adolescents experiencing specific mental health difficulties are required.

## Electronic supplementary material

Below is the link to the electronic supplementary material.

Supplementary material 1 (PDF 157 kb)

Supplementary material 2 (PDF 98 kb)

Supplementary material 3 (PDF 95 kb)

Supplementary material 4 (PDF 114 kb)

Supplementary material 5 (PDF 192 kb)

## References

[1] Polanczyk GV, Salum GA, Sugaya LS (2015). Annual research review: a meta-analysis of the worldwide prevalence of mental disorders in children and adolescents. J Child Psychol Psychiatry Allied Discip.

[2] Green H, McGinnity A, Meltzer H (2005). Mental health of children and young people in Great Britain, 2004.

[3] Pompili M, Serafini G, Innamorati M (2012). Substance abuse and suicide risk among adolescents. Eur Arch Psychiatry Clin Neurosci.

[4] Riegler A, Völkl-Kernstock S, Lesch O (2017). Attention deficit hyperactivity disorder and substance abuse: an investigation in young Austrian males. J Affect Disord.

[5] Ford T, Collishaw S, Meltzer H, Goodman R (2007). A prospective study of childhood psychopathology: independent predictors of change over 3 years. Soc Psychiatry Psychiatr Epidemiol.

[6] Kim-Cohen J, Caspi A, Moffitt TE (2003). Prior juvenile diagnoses in adults with mental disorder: developmental follow-back of a prospective-longitudinal cohort. Arch Gen Psychiatry.

[7] Prince M, Patel V, Saxena S (2007). No health without mental health. Lancet.

[8] Reynolds S, Wilson C, Austin J, Hooper L (2012). Effects of psychotherapy for anxiety in children and adolescents: a meta-analytic review. Clin Psychol Rev.

[9] Sadler K, Ti Vizard, Ford T (2018). Mental health of children and young people in England, 2017.

[10] Chavira DA, Garland A, Yeh M (2009). Child anxiety disorders in public systems of care: comorbidity and service utilization. J Behav Heal Serv Res.

[11] Chavira DA, Stein MB, Bailey K, Stein MT (2004). Child anxiety in primary care: prevalent but untreated. Depress Anxiety.

[12] Reardon T, Harvey K, Baranowska M (2017). What do parents perceive are the barriers and facilitators to accessing psychological treatment for mental health problems in children and adolescents? A systematic review of qualitative and quantitative studies. Eur Child Adolesc Psychiatry.

[13] O’Brien D, Harvey K, Howse J (2016). Barriers to managing child and adolescent mental health problems: a systematic review of primary care practitioners’ perceptions. Br J Gen Pract.

[14] Gulliver A, Griffiths KM, Christensen H (2010). Perceived barriers and facilitators to mental health help-seeking in young people: a systematic review. BMC Psychiatry.

[15] Moore A, Gammie J (2018) Revealed: hundreds of children wait more than a year for specialist help. Health Service J. https://www.hsj.co.uk/quality-and-performance/revealed-hundreds-of-children-wait-more-than-a-year-for-specialist-help/7023232.article. Accessed 15 Sept 2018

[16] Department of Health, Department of Education (2017) Transforming children and young people’s mental health provision: a green paper. Crown Copyright. https://assets.publishing.service.gov.uk/government/uploads/system/uploads/attachment_data/file/664855/Transforming_children_and_young_people_s_mental_health_provision.pdf. Accessed 15 Sept 2018

[17] Department of Health, Department of Education (2018) Government response to the consultation on transforming children and young people’s mental health provision: a green paper and next steps. Crown Copyright. https://assets.publishing.service.gov.uk/government/uploads/system/uploads/attachment_data/file/728892/government-response-to-consultation-on-transforming-children-and-young-peoples-mental-health.pdf. Accessed 15 Sept 2018

[18] Moher D, Liberati A, Tetzlaff J, Altman DG (2009). Systematic reviews and meta-analyses: the PRISMA statement. Annu Intern Med.

[19] Fortune S, Sinclair J, Hawton K (2008). Adolescents’ views on preventing self-harm. Soc Psychiatry Psychiatr Epidemiol.

[20] Fortune S, Sinclair J, Hawton K (2008). Help-seeking before and after episodes of self-harm: a descriptive study in school pupils in England. BMC Public Health.

[21] Lindsey MA, Korr WS, Broitman M (2006). Help-seeking behaviors and depression among African–American adolescent boys. Soc Work.

[22] Lindsey MA, Joe S, Nebbitt V (2010). Family matters: the role of mental health stigma and social support on depressive symptoms and subsequent help seeking among African–American boys. J Black Psychol.

[23] Kmet LM, Lee RC, Cook LS (2004) Standard quality assessment criteria for evaluating primary research papers from a variety of fields. Alberta Heritage Foundation for Medical Research (AHFMR), Edmonton

[24] Meredith LS, Stein BD, Paddock SM (2009). Perceived barriers to treatment for adolescent depression. Med Care.

[25] Popay J, Roberts H, Sowden A (2006). Narrative synthesis in systematic reviews: a product from the ESRC methods programme. ESRC Methods Program.

[26] Boyd CP, Hayes L, Nurse S (2011). Preferences and intention of rural adolescents toward seeking help for mental health problems. Rural Remote Health.

[27] Freedenthal S, Stiffman AR (2007). “They might think i was crazy”: young American Indians’ reasons for not seeking help when suicidal. J Adolesc Res.

[28] Wilson CJ, Rickwood D, Deane FP (2007). Depressive symptoms and help-seeking intentions in young people. Clin Psychol.

[29] D’Amico EJ, McCarthy DM, Metrik J, Brown SA (2004). Alcohol-related services: prevention, secondary intervention, and treatment preferences of adolescents. J Child Adolesc Subst Abuse.

[30] Wilson CJ, Deane FP, Marshall KL, Dalley A (2008). Reducing adolescents’ perceived barriers to treatment and increasing help-seeking intentions: effects of classroom presentations by general practitioners. J Youth Adolesc.

[31] Bussing R, Koro-Ljungberg M, Nohuchi K (2012). Willingness to use ADHD treatments: a mixed methods study of perceptions by adolescents, parents, health professionals and teachers regina. Soc Sci Med.

[32] Chandra A, Minkovitz CS (2006). Stigma starts early: Gender differences in teen willingness to use mental health services. J Adolesc Heal.

[33] Cigularov K, Chen PY, Thurber BW, Stallones L (2008). What prevents adolescents from seeking help after a suicide education program?. Suicide Life-Threat Behav.

[34] Gould MS, Greenberg T, Munfakh JLH (2006). Teenagers’ attitudes about seeking help from telephone crisis services (hotlines). Suicide Life-Threat Behav.

[35] Gould MS, Marrocco FA, Hoagwood K (2009). Service use by at-risk youths after school-based suicide screening. J Am Acad Child Adolesc Psychiatry.

[36] Guo S, Kataoka SH, Bear L, Lau AS (2014). Differences in school-based referrals for mental health care: understanding racial/ethnic disparities between Asian–American and Latino youth. School Ment Health.

[37] Guterman NB, Haj-Yahia MM, Vorhies V (2010). Help-seeking and internal obstacles to receiving support in the wake of community violence exposure: the case of Arab and Jewish Adolescents in Israel. J Child Fam Stud.

[38] Haavik L, Joa I, Hatloy K (2017). Help seeking for mental health problems in an adolescent population: the effect of gender. J Ment Heal.

[39] Khairani O, Zaiton S, Faridah MN (2005). Do adolescents attending Bandar Mas primary care clinic consult health professionals for their common health problems?. Med J Malaysia.

[40] Kuhl J, Jarkon-Horlick L, Morrissey RF (1997). Measuring barriers to help-seeking behavior in adolescents. J Youth Adolesc.

[41] Lubman DI, Cheetham A, Jorm AF (2017). Australian adolescents’ beliefs and help-seeking intentions towards peers experiencing symptoms of depression and alcohol misuse. BMC Public Health.

[42] Muthupalaniappen L, Omar J, Omar K (2012). Emotional and behavioral problems among adolescent smokers and their help-seeking behavior. Southeast Asian J Trop Med Public Health.

[43] Samargia LA, Saewyc EM, Elliott BA (2006). Foregone mental health care and self-reported access barriers among adolescents. J Sch Nurs.

[44] Sharma M, Banerjee B, Garg S (2017). Assessment of mental health literacy in school-going adolescents. J Indian Assoc Child Adolesc Ment Heal.

[45] Sheffield JK, Fiorenza E, Sofronoff K (2004). Adolescents’ willingness to seek psychological help: promoting and preventing factors. J Youth Adolesc.

[46] Sylwestrzak A, Overholt CE, Ristau KI, Coker KL (2015). Self-reported barriers to treatment engagement: adolescent perspectives from the National Comorbidity Survey-Adolescent Supplement (NCS-A). Community Ment Health J.

[47] Wilson CJ, Deane FP (2012). Brief report: Need for autonomy and other perceived barriers relating to adolescents’ intentions to seek professional mental health care. J Adolesc.

[48] Wu MS, Salloum A, Lewin AB (2016). Treatment concerns and functional impairment in pediatric anxiety. Child Psychiatry Hum Dev.

[49] Balle Tharaldsen K, Stallard P, Cuijpers P (2017). ‘It’s a bit taboo’: a qualitative study of Norwegian adolescents’ perceptions of mental healthcare services. Emot Behav Difficulties.

[50] Becker SJ, Swenson RR, Esposito-Smythers C (2014). Barriers to seeking mental health services among adolescents in military families. Prof Psychol Res Pract.

[51] Breland-Noble AM, Wong MJ, Childers T (2015). Spirituality and religious coping in African–American youth with depressive illness. Ment Heal Relig Cult.

[52] Bullock M, Nadeau L, Renaud J (2012). Spirituality and religion in youth suicide attempters’ trajectories of mental health service utilization: the year before a suicide attempt. J Can Acad Child Adolesc Psychiatry.

[53] Chandra A, Minkovitz CS (2007). Factors that influence mental health stigma among 8th grade adolescents. J Youth Adolesc.

[54] Clark LH, Hudson JL, Dunstan DA, Clark GI (2018). Barriers and facilitating factors to help-seeking for symptoms of clinical anxiety in adolescent males. Aust J Psychol.

[55] De Anstiss H, Ziaian T (2010). Mental health help-seeking and refugee adolescents: qualitative findings from a mixed-methods investigation. Aust Psychol.

[56] Del Mauro JM, Jackson Williams D (2013). Children and adolescents’ attitudes toward seeking help from professional mental health providers. Int J Adv Couns.

[57] Doyle L, Treacy MP, Sheridan A (2017). ‘It just doesn’t feel right’: a mixed methods study of help-seeking in Irish schools. Adv Sch Ment Health Promot.

[58] Fleming TM, Dixon RS, Merry SN (2012). “It’s mean!” the views of young people alienated from mainstream education on depression, help seeking and computerised therapy. Adv Ment Heal.

[59] Fornos LB, Mika VS, Bayles B (2005). A qualitative study of Mexican–American adolescents and depression. J Sch Health.

[60] Francis K, Boyd C, Aisbett D (2006). Rural adolescents’ attitudes to seeking help for mental health problems. Youth Stud Aust.

[61] Gonçalves M, Moleiro C, Goncalves M, Moleiro C (2012). The family–school–primary care triangle and the access to mental health care among migrant and ethnic minorities. J Immigr Minor Heal.

[62] Gronholm PC, Thornicroft G, Laurens KR, Evans-Lacko S (2017). Conditional disclosure on pathways to care: coping preferences of young people at risk of psychosis. Qual Health Res.

[63] Hassett A, Isbister C (2017). Young men’s experiences of accessing and receiving help from child and adolescent mental health services following self-harm. SAGE Open.

[64] Huggins A, Weist MD, McCall M (2016). Qualitative analysis of key informant interviews about adolescent stigma surrounding use of school mental health services. Int J Ment Health Promot.

[65] Kendal S, Keeley P, Callery P (2014). Student help seeking from pastoral care in UK high schools: a qualitative study. Child Adolesc Ment Health.

[66] Klineberg E, Kelly MJ, Stansfeld SA, Bhui KS (2013). How do adolescents talk about self-harm: a qualitative study of disclosure in an ethnically diverse urban population in England. BMC Public Health.

[67] Leavey G, Rothi D, Paul R (2011). Trust, autonomy and relationships: the help-seeking preferences of young people in secondary level schools in London (UK). J Adolesc.

[68] Lindsey MA, Chambers K, Pohle C (2013). Understanding the behavioral determinants of mental health service use by urban, under-resourced black youth: adolescent and caregiver perspectives. J Child Fam Stud.

[69] McAndrew S, Warne T (2014). Hearing the voices of young people who self-harm: implications for service providers. Int J Ment Health Nurs.

[70] Mueller AS, Abrutyn S (2016). Adolescents under pressure: a new durkheimian framework for understanding adolescent suicide in a Cohesive Community. Am Sociol Rev.

[71] Pailler ME, Cronholm PF, Barg FK (2009). Patients’ and caregivers’ beliefs about depression screening and referral in the Emergency Department. Pediatr Emerg Care.

[72] Prior S (2012). Young people’s process of engagement in school counselling. Couns Psychother Res.

[73] Timlin-Scalera RM, Ponterotto JG, Blumberg FC, Jackson MA (2003). A grounded theory study of help-seeking behaviors among white male high school students. J Couns Psychol.

[74] Wilson CJ, Deane FP (2001). Adolescent opinions about reducing help-seeking barriers and increasing appropriate help engagement. J Educ Psychol Consult.

[75] Wisdom JP, Clarke GN, Green CA (2006). What teens want: barriers to seeking care for depression. Adm Policy Ment Heal Ment Heal Serv Res.

[76] Mellor C (2014). School-based interventions targeting stigma of mental illness: systematic review. Psychiatr Bull.

[77] Wolpert M, Cortina M (2018). CASCADE Framework: supporting joint working between education and mental health professionals.

[78] Fazel M, Hoagwood K, Stephan S, Ford T (2014). Mental health interventions in schools 1: mental health interventions in schools in high-income countries. lancet Psychiatry.

