# Supplementary material for: Why do children and adolescents (not) seek and access professional help for their mental health problems? A systematic review of quantitative and qualitative studies
Source: Eur Child Adolesc Psychiatry. 2020 Jan 21;30(2):183–211. doi: 10.1007/s00787-019-01469-4 (PMC7932953; doi:10.1007/s00787-019-01469-4)
Supplement: Supplementary file 2 — Supplementary material 2 (PDF 98 kb) [file 787_2019_1469_MOESM2_ESM.pdf]

Why do children and adolescents (not) seek and access professional help for their mental health problems? ***A systematic review of quantitative and qualitative studies***

European Child and Adolescent Psychiatry

Jerica Radez[1], Tessa Reardon[1,2], Cathy Creswell[2], Peter J Lawrence[3], Georgina Evdoka-Burton[4], Polly Waite[1,2]

[1] School of Psychology and Clinical Language Sciences, University of Reading, Reading, UK

[2] Departments of Experimental Psychology and Psychiatry, University of Oxford, Oxford, UK

[3] School of Psychology, University of Southampton, Southampton, UK

[4] Slough Community Mental Health Team, Berkshire Healthcare NHS Foundation Trust, Slough, UK

*Correspondence to Dr Tessa Reardon (tessa.reardon@psych.ox.ac.uk)

Electronic Supplementary Material 2: Literature Search Strategy

The search terms captured four major theoretical concepts (see Table 1 for full search terms): barriers/facilitators; help seeking; mental health; parents/children and adolescents. The search terms for each of these concepts were generated and combined using 'AND' to search for titles and articles. In addition, we used hand search methods to check the reference list of articles included in the full text screening stage to identify further studies of interest.

Table 1 Search terms

Group 1	Group 2	Group 3	Group 4
Barrier*	Help-seeking	Mental*	Child*
Hurdle	help seeking	disorder*	Adolescen*
Obstruct*	helpseek*	Psychopathology	Youth
Obstacle	'Seek* help'	'Emotional problems'	Parent*
Promot*	'Care seeking'	'Emotional distress'	Caregiver*
Facilitat*	'seek* care'	'Behavio* problems'	Carer*
Encourage*	'access treatment'	Internali*	Teen*
Support*	'access service'	Anxi*	Mother*
Cause*	'seek* treatment'	Affect*	Father*
Predict*	'Service* use'	Depress*	Maternal
'Unmet need'	'Service* utilisation'	Suicid*	Paternal
Hinder	'Service* utilization'	Externali*	
Willingness	'treatment participation'	ADHD	
Enable*	'treatment engagement'	'self harm'	
		Psychosis	
		Oppositional	
		Conduct	
