# Supplementary material for: Why do children and adolescents (not) seek and access professional help for their mental health problems? A systematic review of quantitative and qualitative studies
Source: Eur Child Adolesc Psychiatry. 2020 Jan 21;30(2):183–211. doi: 10.1007/s00787-019-01469-4 (PMC7932953; doi:10.1007/s00787-019-01469-4)
Supplement: Supplementary file 3 — Supplementary material 3 (PDF 95 kb) [file 787_2019_1469_MOESM3_ESM.pdf]

Why do children and adolescents (not) seek and access professional help for their mental health problems? ***A systematic review of quantitative and qualitative studies***

European Child and Adolescent Psychiatry

Jerica Radez[1], Tessa Reardon[1,2], Cathy Creswell[2], Peter J Lawrence[3], Georgina Evdoka-Burton[4], Polly Waite[1,2]

[1] School of Psychology and Clinical Language Sciences, University of Reading, Reading, UK

[2] Departments of Experimental Psychology and Psychiatry, University of Oxford, Oxford, UK

[3] School of Psychology, University of Southampton, Southampton, UK

[4] Slough Community Mental Health Team, Berkshire Healthcare NHS Foundation Trust, Slough, UK

*Correspondence to Dr Tessa Reardon (tessa.reardon@psych.ox.ac.uk)

Electronic Supplementary Material 3: Full list of eligibility criteria

A study was selected for inclusion if:

1) Participants were children/adolescents.
2) Children's/Adolescents' perceived barriers/facilitators to accessing professional help for their mental health problems were reported.
3) Study addressed mental health problems; emotional and/or behavioural problems; or a specific mental health disorder.
4) Study was published in English in a peer-reviewed journal.

A study was excluded if:

1) Study did not report barriers/facilitators to accessing treatment for mental health problems in children/adolescents.
2) Study was a review or a theoretical article.
3) Study was not published in English in a peer-reviewed journal.
4) Study only reported parental/caregiver's perceived barriers/facilitators to accessing treatment for mental health problems.
5) Study only reported factors associated with or predictors of parent or child/adolescent help seeking behaviour/service use/help seeking intentions.
6) Study only reported findings from an intervention designed to address one or more barrier to help-seeking.
7) Study only reported perceived barriers/facilitators to accessing ongoing to treatment, not *initial* access to treatment.
8) Study only reported perceived barriers/facilitators to accessing treatment for autism spectrum disorder or developmental disabilities.
9) Participants in the study were not children or adolescents.
10) The mean age of the children/adolescents was >18 years or if the sample included adults >21 years.
11) Study did not report any qualitative or quantitative data that could be extracted.
12) Study only reported perceived barriers/facilitators to accessing medication or inpatient psychiatric care.
13) Participants in the study represented a special population (i.e., children/adolescents with an intellectual or developmental disability, young offenders, children/adolescents with mental health problems in the context of a specific physical health condition, looked after children).
14) Study only reported perceived barriers/facilitators to accessing parenting support not specifically targeted at mental health problems in children.
