# Supplementary material for: Why do children and adolescents (not) seek and access professional help for their mental health problems? A systematic review of quantitative and qualitative studies
Source: Eur Child Adolesc Psychiatry. 2020 Jan 21;30(2):183–211. doi: 10.1007/s00787-019-01469-4 (PMC7932953; doi:10.1007/s00787-019-01469-4)
Supplement: Supplementary file 4 — Supplementary material 4 (PDF 114 kb) [file 787_2019_1469_MOESM4_ESM.pdf]

Why do children and adolescents (not) seek and access professional help for their mental health problems? ***A systematic review of quantitative and qualitative studies***

European Child and Adolescent Psychiatry

Jerica Radez[1], Tessa Reardon[1,2], Cathy Creswell[2], Peter J Lawrence[3], Georgina Evdoka-Burton[4], Polly Waite[1,2]

[1] School of Psychology and Clinical Language Sciences, University of Reading, Reading, UK

[2] Departments of Experimental Psychology and Psychiatry, University of Oxford, Oxford, UK

[3] School of Psychology, University of Southampton, Southampton, UK

[4] Slough Community Mental Health Team, Berkshire Healthcare NHS Foundation Trust, Slough, UK

*Correspondence to Dr Tessa Reardon (tessa.reardon@psych.ox.ac.uk)

Electronic Supplementary Material 4: Quality Rating Checklists

Table 1 Quality rating checklist for quantitative studies

	Yes = 2	Partial = 1	No = 0
Question / objective sufficiently described			
Study design evident and appropriate			
Method of participant selection described and appropriate			
Sample size appropriate			
Participant characteristics sufficiently described			
Measure of barriers/facilitators well defined			
Measure robust			
Analyses described/justified and appropriate			
Results reported in sufficient detail			
Conclusions supported by the results			

Modified version of Kmet et al's (2004) Standard Quality Assessment Criteria

Table 2 Quality rating checklist for qualitative studies

	Yes = 2	Partial = 1	No = 0
Question/objective sufficiently described			
Are the research question/s suited to qualitative inquiry			
Study design well described and appropriate			
Context of study clear			
Sampling strategy described, appropriate and justified			
Data collection methods systematic, clearly described and appropriate for research question			
Data analysis systematic, clearly described and appropriate for research question			
Use of verification procedures to establish credibility			
Are the claims/conclusion credible?			

Modified version of Kmet et al's (2004) Standard Quality Assessment Criteria, incorporating Dixon-Woods et al's (2004) prompts
