# Supplementary material for: Why do children and adolescents (not) seek and access professional help for their mental health problems? A systematic review of quantitative and qualitative studies
Source: Eur Child Adolesc Psychiatry. 2020 Jan 21;30(2):183–211. doi: 10.1007/s00787-019-01469-4 (PMC7932953; doi:10.1007/s00787-019-01469-4)
Supplement: Supplementary file 5 — Supplementary material 5 (PDF 192 kb) [file 787_2019_1469_MOESM5_ESM.pdf]

Why do children and adolescents (not) seek and access professional help for their mental health problems? ***A systematic review of quantitative and qualitative studies***

European Child and Adolescent Psychiatry

Jerica Radez[1], Tessa Reardon[1,2], Cathy Creswell[2], Peter J Lawrence[3], Georgina Evdoka-Burton[4], Polly Waite[1,2]

[1] School of Psychology and Clinical Language Sciences, University of Reading, Reading, UK

[2] Departments of Experimental Psychology and Psychiatry, University of Oxford, Oxford, UK

[3] School of Psychology, University of Southampton, Southampton, UK

[4] Slough Community Mental Health Team, Berkshire Healthcare NHS Foundation Trust, Slough, UK

*Correspondence to Dr Tessa Reardon (tessa.reardon@psych.ox.ac.uk)

Electronic Supplementary Material 5: List of barrier and facilitator themes and subthemes identified in each study

Table 1 Barriers and facilitators identified in quantitative studies

	Barrier/Facilitator themes and subthemes identified			
Study	Young people's individual factors	Social factors	Relationship factors	Systemic and structural factors
Boyd et al. (2011)	Past experiences, Preference for informal support, Preference for self-reliance	Views and attitudes towards MH and help-seeking in the wider environment	Ability to trust unknown person	Accessibility of professional help, Availability of professional help, Logistical factors
Chandra & Minkovitz (2006)	Knowledge about MH and MH services, Perceived effectiveness of professional help, Ability to verbalise the need for help and to talk about MH difficulties	Views and attitudes towards MH and help-seeking in the wider environment, Views and attitudes towards MH and help-seeking within YP's support network	Ability to trust unknown person	
Cigularov, Chen, Thurber, & Stallones (2008)	Broader perceptions of help-seeking, Perceived effectiveness of professional help, Emotional and motivational factors, Factors associated with commitment to the process of help-seeking	Views and attitudes towards MH and help-seeking in the wider environment, Anticipated consequences of help-seeking on social network	Ability to trust unknown person, Perceived confidentiality	Accessibility of professional help
D'Amico et al. (2004)		Views and attitudes towards MH and help-seeking within YP's support network	Perceived confidentiality, Perception of contact with professionals, Similarity between YP and MH professional	Logistical factors
Freedenthal & Stiffman (2007)	Knowledge about MH and MH services, Broader perceptions of help-seeking, Emotional and motivational factors, Preference for informal support, Preference for self-reliance	Views and attitudes towards MH and help-seeking in the wider environment		
Gould et al. (2006)	Knowledge about MH and MH services, Perceived effectiveness of professional help, Factors associated with commitment to the process of help-seeking, Ability to verbalise the need for help and to talk about MH difficulties, Preference for informal support, Preference for self-reliance	Views and attitudes towards MH and help-seeking in the wider environment		
Gould et al. (2009)	Knowledge about MH and MH services, Factors associated with commitment to the process of help-seeking, Past experiences		Ability to trust unknown person	Costs of professional help, Logistical factors
Guo, Kataoka, Bear, & Lau (2014)	Knowledge about MH and MH services, Ability to verbalise the need for help and to talk about MH difficulties	Views and attitudes towards MH and help-seeking in the wider environment	Perceived confidentiality, Similarity between YP and MH professional	Costs of professional help, Logistical factors
Guterman, Haj-Yahia, Vorhies, Ismayilova, & Leshem (2010)	Knowledge about MH and MH services, Preference for self-reliance, Broader perceptions of help-seeking, Perceived effectiveness of professional help, Factors associated with commitment to the process of help-seeking, Ability to verbalise the need for help and to talk about MH difficulties	Views and attitudes towards MH and help-seeking in the wider environment, Views and attitudes towards MH and help-seeking within YP's support network	Perceived confidentiality, Perception of contact with professionals	

Haavik, Joa, Hatloy, Stain, & Langeveld, (2017)	Knowledge about MH and MH services, Broader perceptions of help-seeking, Factors associated with commitment to the process of help-seeking		Perception of contact with professionals	Availability of professional help, Costs of professional help
Khairani, Zaiton, & Faridah (2005)	Knowledge about MH and MH services, Emotional and motivational factors, Ability to verbalise the need for help and to talk about MH difficulties		Perceived confidentiality	
Kuhl, Jarkon-Horlick, & Morrissey (1997)	Knowledge about MH and MH services, Broader perceptions of help-seeking, Perceived effectiveness of professional help, Emotional and motivational factors, Factors associated with commitment to the process of help-seeking, Past experiences, Perceived autonomy, Preference for informal support, Preference for self-reliance	Views and attitudes towards MH and help-seeking in the wider environment, Views and attitudes towards MH and help-seeking within YP's support network, Anticipated consequences of help-seeking on social network	Similarity between YP and MH professional	Costs of professional help, Logistical factors
Lubman et al. (2017)	Preference for self-reliance, Perceived autonomy, Emotional and motivational factors	Views and attitudes towards MH and help-seeking in the wider environment		Costs of professional help
Meredith et al. (2009)	Preference for self-reliance	Views and attitudes towards MH and help-seeking in the wider environment, Views and attitudes towards MH and help-seeking within YP's support network		Accessibility of professional help, Availability of professional help, Costs of professional help, Logistical factors
Muthupalaniappen et al. (2012)	Knowledge about MH and MH services, Perceived effectiveness of professional help, Factors associated with commitment to the process of help-seeking, Preference for informal support, Preference for self-reliance	Views and attitudes towards MH and help-seeking in the wider environment	Ability to trust unknown person	Costs of professional help, Logistical factors
Samargia, Saewyc, & Elliott (2006)	Knowledge about MH and MH services, Factors associated with commitment to the process of help-seeking, Perceived autonomy, Emotional and motivational factors	Views and attitudes towards MH and help-seeking in the wider environment		Costs of professional help, Logistical factors
Sharma, Banerjee, & Garg (2017)	Knowledge about MH and MH services, Perceived effectiveness of professional help	Views and attitudes towards MH and help-seeking in the wider environment		
Sheffield, Fiorenza, & Sofronoff (2004)	Knowledge about MH and MH services, Perceived effectiveness of professional	Views and attitudes towards MH and help-seeking in the wider environment	Perceived confidentiality	Costs of professional help, Logistical factors

	help, Perceived autonomy, Preference for self-reliance			
Sylwestrzak, Overholt, Ristau, & Coker (2015)	Knowledge about MH and MH services, Broader perceptions of help-seeking, Perceived effectiveness of professional help, Factors associated with commitment to the process of help-seeking, Past experiences, Preference for self-reliance	Views and attitudes towards MH and help-seeking in the wider environment		Costs of professional help, Logistical factors
Wilson & Deane (2012)	Broader perceptions of help-seeking, Emotional and motivational factors, Past experiences, Perceived autonomy, Preference for self-reliance	Views and attitudes towards MH and help-seeking in the wider environment	Perceived confidentiality, Similarity between YP and MH professional	
Wilson et al. (2007)	Preference for self-reliance	Views and attitudes towards MH and help-seeking in the wider environment	Ability to trust unknown person	
Wilson et al. (2008)	Knowledge about MH and MH services, Factors associated with commitment to the process of help-seeking, Perceived autonomy	Views and attitudes towards MH and help-seeking in the wider environment	Perception of contact with professionals	
Wu et al. (2016)	Broader perceptions of help-seeking, Perceived effectiveness of professional help, Factors associated with commitment to the process of help-seeking, Ability to verbalise the need for help and to talk about MH difficulties, Perceived autonomy	Views and attitudes towards MH and help-seeking in the wider environment, Views and attitudes towards MH and help-seeking within YP's support network, Anticipated consequences of help-seeking on social network		Logistical factors

Table 2 Barriers and facilitators identified in qualitative studies

	Barrier/Facilitator themes and subthemes identified			
Study	Young people's individual factors	Social factors	Relationship factors	Systemic and structural factors
Balle Tharaldsen, Stallard, Cuijpers, Bru, & Bjaastad (2017)	Broader perceptions of help-seeking, Ability to verbalise the need for help and to talk about MH difficulties	Views and attitudes towards MH and help-seeking in the wider environment, Anticipated consequences of help-seeking on social network		Logistical factors
Becker, Swenson, Esposito-Smythers, Cataldo, & Spirito (2014)	Knowledge about MH and MH services, Perceived effectiveness of professional help, Preference for self-reliance	Views and attitudes towards MH and help-seeking in the wider environment	Perceived confidentiality	Costs of professional help, Logistical factors
Breland-Noble, Wong, Childers, Hankerson, & Sotomayor (2015)		Views and attitudes towards MH and help-seeking in the wider environment		
Bullock, Nadeau, & Renaud (2012)	Preference for informal support	Views and attitudes towards MH and help-seeking within YP's support network		
Bussing et al. (2012)	Broader perceptions of help-seeking, Perceived effectiveness of professional help	Views and attitudes towards MH and help-seeking in the wider environment, Anticipated consequences of help-seeking on social network		Costs of professional help
Chandra & Minkovitz (2007)	Broader perceptions of help-seeking, Past experiences	Views and attitudes towards MH and help-seeking within YP support network, Anticipated consequences of help-seeking on social network		
Clark, Hudson, Dunstan, & Clark (2018)	Knowledge about MH and MH services, Broader perceptions of help-seeking, Emotional and motivational factors, Preference for self-reliance	Views and attitudes towards MH and help-seeking in the wider environment		Accessibility of professional help, Availability of professional help, Use of information technology
De Anstiss & Ziaian (2010)	Knowledge about MH and MH services, Broader perceptions of help-seeking, Factors associated with commitment to the process of help-seeking, Preference for informal support	Views and attitudes towards MH and help-seeking in the wider environment, Anticipated consequences of help-seeking on social network	Similarity between YP and MH professional	
Del Mauro & Jackson Williams (2013)	Factors associated with commitment to the process of help-seeking, Ability to verbalise the need for help and to talk about MH difficulties, Preference for informal support, Preference for self-reliance	Views and attitudes towards MH and help-seeking in the wider environment	Perceived confidentiality, Perception of contact with professionals	
Doyle, Treacy, & Sheridan (2017)	Knowledge about MH and MH services, Perceived autonomy	Views and attitudes towards MH and help-seeking in the wider environment	Ability to trust unknown person, Perceived confidentiality	Availability of professional help

Fleming, Dixon, & Merry (2012)	Emotional and motivational factors	Views and attitudes towards MH and help-seeking in the wider environment	Ability to trust unknown person	Use of information technology
Fornos et al. (2005)	Knowledge about MH and MH services, Broader perceptions of help-seeking	Views and attitudes towards MH and help-seeking in the wider environment, Views and attitudes towards MH and help-seeking within YP's support network, Anticipated consequences of help-seeking on social network	Perceived confidentiality	Costs of professional help
Fortune et al. (2008a); Fortune et al. (2008b)	Knowledge about MH and MH services, Broader perceptions of help-seeking, Emotional and motivational factors, Ability to verbalise the need for help and to talk about MH difficulties, Preference for informal support, Preference for self-reliance	Views and attitudes towards MH and help-seeking in the wider environment, Views and attitudes towards MH and help-seeking within YP's support network, Anticipated consequences of help-seeking on social network	Perceived confidentiality, Perception of contact with professionals	Accessibility of professional help
Francis, Boyd, Aisbett, Newnham, & Newnham (2006)	Broader perceptions of help-seeking	Views and attitudes towards MH and help-seeking in the wider environment		Availability of professional help, Logistical factors
Gonçalves, Moleiro, Goncalves, & Moleiro (2012)	Perceived autonomy, Preference for informal support, Preference for self-reliance	Views and attitudes towards MH and help-seeking in the wider environment, Views and attitudes towards MH and help-seeking within YP's support network	Similarity between YP and MH professional	Costs of professional help
Gronholm, Thornicroft, Laurens, & Evans-Lacko (2017)	Factors associated with commitment to the process of help-seeking	Views and attitudes towards MH and help-seeking in the wider environment	Perceived confidentiality	
Hassett & Isbister (2017)	Knowledge about MH and MH services, Broader perceptions of help-seeking, Factors associated with commitment to the process of help-seeking, Ability to verbalise the need for help and to talk about MH difficulties, Perceived autonomy	Views and attitudes towards MH and help-seeking in the wider environment, Views and attitudes towards MH and help-seeking within YP's support network	Perception of contact with professionals, Similarity between YP and MH professional	Use of information technology, Logistical factors
Huggins et al. (2016)	Knowledge about MH and MH services	Views and attitudes towards MH and help-seeking in the wider environment	Perceived confidentiality	Accessibility of professional help
Kendal, Keeley, & Callery (2014)	Knowledge about MH and MH services, Factors associated with commitment to the process of help-seeking, Perceived autonomy	Anticipated consequences of help-seeking on social network	Perceived confidentiality, Ability to trust unknown person	Accessibility of professional help
Klineberg, Kelly, Stansfeld, & Bhui (2013)	Factors associated with commitment to the process of help-seeking	Views and attitudes towards MH and help-seeking in the wider environment, Anticipated consequences of help-seeking on social network	Perceived confidentiality, Perception of contact with professionals, Similarity between YP and MH professional	

Leavey, Rothi, & Paul (2011)	Perceived effectiveness of professional help, Perceived autonomy		Perception of contact with professionals	Accessibility of professional help, Availability of professional help, Use of information technology
Lindsey, Chambers, Pohle, Beall, & Lucksted (2013)	Broader perceptions of help-seeking, Ability to verbalise the need for help and to talk about MH difficulties, Preference for informal support, Preference for self-reliance	Views and attitudes towards MH and help-seeking in the wider environment, Views and attitudes towards MH and help-seeking within YP's support network, Anticipated consequences of help-seeking on social network	Ability to trust unknown person, Similarity between YP and MH professional	Availability of professional help
Lindsey et al. (2006); Lindsey, Joe, & Nebbitt (2010)	Broader perceptions of help-seeking, Preference for informal support, Preference for self-reliance	Views and attitudes towards MH and help-seeking in the wider environment, Views and attitudes towards MH and help-seeking within YP's support network	Ability to trust unknown person, Similarity between YP and MH professional	
Mcandrew & Warne (2014)	Knowledge about MH and MH services, Emotional and motivational factors	Views and attitudes towards MH and help-seeking in the wider environment	Perceived confidentiality, Perception of contact with professionals	Accessibility of professional help
Meredith et al. (2009)	Factors associated with commitment to the process of help-seeking	Views and attitudes towards MH and help-seeking in the wider environment		Logistical factors
Mueller & Abrutyn (2016)		Views and attitudes towards MH and help-seeking in the wider environment		
Pailler et al. (2009)	Factors associated with commitment to the process of help-seeking	Views and attitudes towards MH and help-seeking within YP's support network		Costs of professional help, Logistical factors
Prior (2012)	Perceived effectiveness of professional help, Perceived autonomy	Views and attitudes towards MH and help-seeking in the wider environment, Views and attitudes towards MH and help-seeking within YP's support network	Ability to trust unknown person	
Timlin-Scalera, Ponterotto, Blumberg, & Jackson (2003)	Knowledge about MH and MH services, Broader perceptions of help-seeking, Factors associated with commitment to the process of help-seeking, Preference for self-reliance, Past experiences	Views and attitudes towards MH and help-seeking in the wider environment, Views and attitudes towards MH and help-seeking within YP's support network	Perceived confidentiality	Availability of professional help
Wilson & Deane (2001)	Knowledge about MH and MH services, Factors associated with commitment to the process of help-seeking, Past experiences	Views and attitudes towards MH and help-seeking in the wider environment	Perceived confidentiality, Ability to trust unknown person, Perception of contact with professionals	
Wisdom, Clarke, & Green (2006)	Broader perceptions of help-seeking, Perceived autonomy	Views and attitudes towards MH and help-seeking in the wider environment		
